# [Corrigendum] Astragaloside IV promotes the proliferation and migration of osteoblast-like cells through the hedgehog signaling pathway

**DOI:** 10.3892/ijmm.2026.5777

**Published:** 2026-03-02

**Authors:** Li-Hua Guo, Yu Cao, Run-Tao Zhuang, Yan Han, Jun Li

Int J Mol Med 43: 830-838, 2019; DOI: 10.3892/ijmm.2018.4013

Following the publication of the above article, an interested reader drew to the authors' attention that, in Fig. 2D on p. 834 showing the results of Transwell cell migration assay experiments for the U-2OS cell line, the 'U-2OS 24 h/Control' and 'U-2OS 24 h/AST-IV' data panels contained an overlapping section, such that these data panels were apparently derived from the same original source, where the results of differently performed experiments were intended to have been portrayed. Upon performing an independent analysis of the data in this paper in the Editorial Office, it also came to light that two pairs of data panels comparing Figs. 2C and 4C, and 2D and 4D, also contained overlapping sections.

After having consulted their original data, the authors realized that Fig. 2 had inadvertently been assembled incorrectly. The revised version of Fig. 2, now showing the correct data for the 'MG-64 48 h/AST-IV', U-2OS 24 h/AST-IV' and 'U-2OS 48 h/AST-IV' panels in Fig. 2C and D, is shown on the next page. The authors can confirm that the errors associated with this figure did not have any significant impact on either the results or the conclusions reported in this study, and all the authors agree with the publication of this Corrigendum. The authors are grateful to the Editor of *International Journal of Molecular Medicine* for allowing them the opportunity to publish this Corrigendum; furthermore, they apologize to the readership of the Journal for any inconvenience caused.

## Figures and Tables

**Figure 2:**
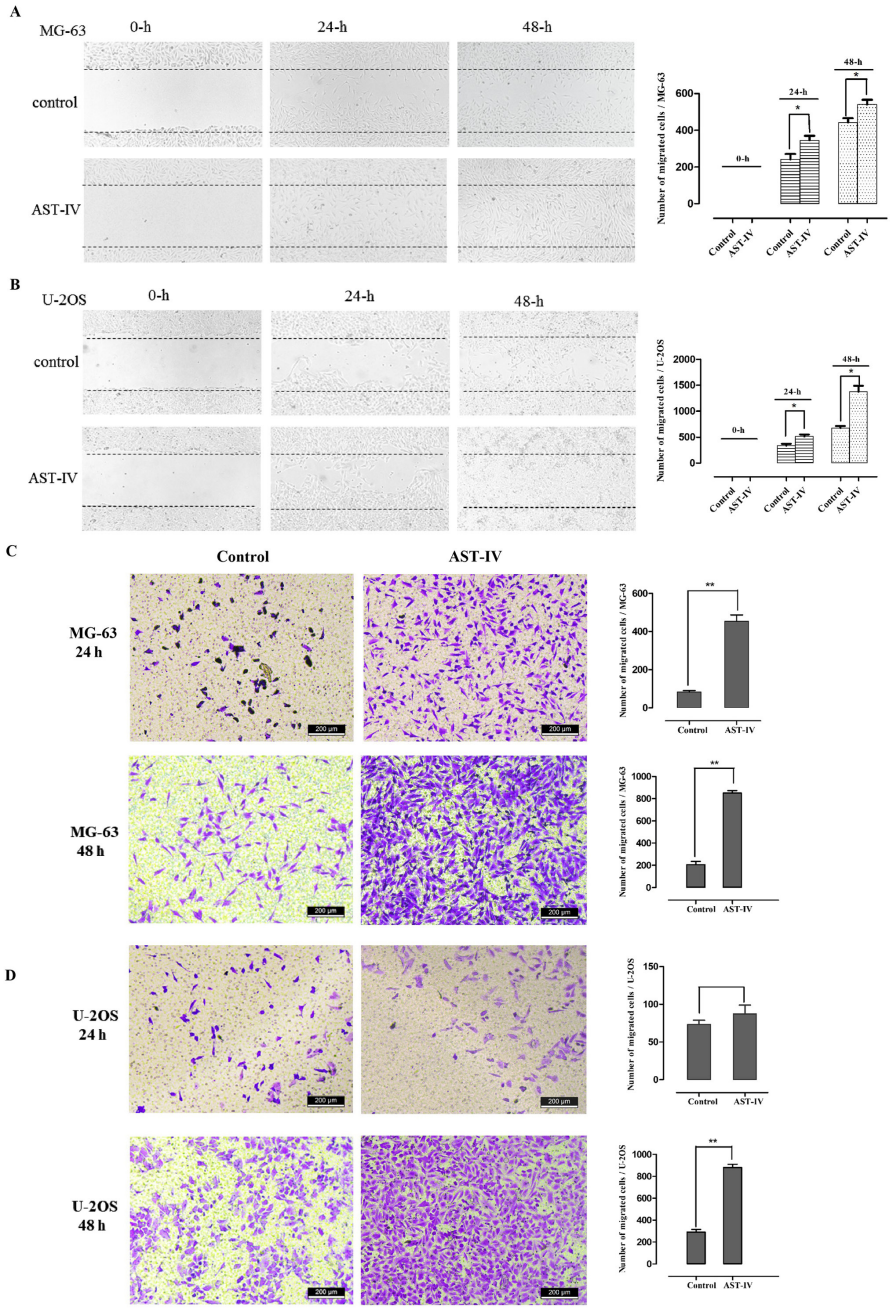
AST-IV increases the migration of MG-63 and U-2OS cells *in vitro*. When the cells had been treated with dimethyl sulfoxide as a control or AST-IV (MG-63, 1×10^−2^ *μ*g/ml; U-2OS, 1×10^−3^ *μ*g/ml) for 48 h, a wound-healing assay for the (A) MG-63 and (B) U-2OS cells, and a Transwell cell migration assay for the (C) MG-63 and (D) U-2OS cells, were performed to evaluate the effects of AST-IV on the metastasis of the cell lines, shown by representative images. The number of migrated cells per high-power field was determined, as shown in the graphs. Data are presented as the mean + standard deviation (n=3), ^*^P<0.05 and ^**^P<0.01, determined by one-way analysis of variance. AST-IV, astragaloside IV.

